# Ag–ZnO Nanocomposites Are Used for SERS Substrates and Promote the Coupling Reaction of PATP

**DOI:** 10.3390/ma14040922

**Published:** 2021-02-15

**Authors:** Liping Ma, Qijia Zhang, Jia Li, Xuemei Lu, Ce Gao, Peng Song, Lixin Xia

**Affiliations:** 1 College of Chemistry, Liaoning University, Shenyang 110036, China; wksy@lnu.edu.cn (L.M.); zqj13940305120@163.com (Q.Z.); gaoce18802427994@163.com (C.G.); 2 College of Physics, Liaoning University, Shenyang 110036, China; lijia@lnu.edu.cn (J.L.); luxuemei@lnu.edu.cn (X.L.); 3 Yingkou Institute of Technology, Yingkou 115014, China

**Keywords:** Ag–ZnO, coupling reaction, nanocomposite, PATP, SERS substrate

## Abstract

Noble metal-semiconductor nanocomposites have received extensive attention in Surface Enhanced Raman Scattering (SERS) due to their unique properties. In this paper, the Ag–ZnO nanocomposites are prepared by hydrothermal growth and simple chemical reduction immersion. The synthesized nanocomposite material simultaneously integrates the individual enhancement effects of the two materials in the SERS, such as the electromagnetic enhancement of silver nanoparticles and the chemical enhancement of ZnO semiconductor materials. Using this substrate, Rhodamine 6G molecules with a concentration as low as 10^−8^ M can be detected, and the coupling reaction of PATP can be effectively promoted. The nanocomposite materials prepared by selecting appropriate semiconductor materials and metal materials combined, could be potentially applied, as SERS substrates, in certain catalytic reactions.

## 1. Introduction

Surface-enhanced Raman scattering (SERS) refers to the phenomenon that the Raman signals of analytes around the metallic nanostructures is amplified by several orders of magnitude owing to the enhancement of the local electromagnetic field induced by the excitation of the surface plasmon resonance [1,2,3,4,5]. Owing to its high sensitivity, SERS is extensively applied in trace molecular detection, biomolecular analysis, and material characterization, besides other fields [6,7,8]. The SERS substrate significantly affects the SERS enhancement effect, and a substantial research has been pooled for finding a type of SERS substrate that has a uniform and stable enhancement effect [9,10].

When light is incident on the metal interface, surface plasmons are generated. This phenomenon is considered to be the primary cause for SERS. Therefore, the noble metal structures have been selected as the best material for SERS substrates [11]. Various semiconductor materials have been detected to produce weak SERS activity enhancement factors of 10^1^–10^3^. The enhancement follows from the chemical enhancement caused by the charge transfer [12,13,14]. Therefore, the combination of semiconductors and metal materials has attracted the attention of researchers, and there have been numerous reports on the application of such composite materials in SERS [15,16,17].

ZnO has received special attention, owing to its excellent performance as a multifunctional semiconductor material, in supporting the chemical enhancement of SERS substrates [18,19]. Furthermore, its composite with precious metal nanoparticles as a SERS substrate has also become a research hotspot. Existing research results show that through the combined effects of electromagnetic enhancement and chemical enhancement, noble metal-ZnO composite nanomaterials can be used as viable materials for SERS enhancement substrates [20,21,22,23,24,25,26]. Here, we have prepared an Ag–ZnO nanocomposite as a SERS substrate, which has excellent reinforcement properties and good uniformity. We have used R6G as the probe molecule to detect the enhancement effect of the composite material as the SERS substrate, and concomitantly, the substrate also has excellent uniformity. Owing to the electron transfer between Ag and ZnO and the local surface plasmon effect of the metal surface, the application of the substrate can effectively promote the catalytic coupling reaction that converts PATP to DMAB. The nanocomposite materials, prepared by selecting the appropriate semiconductor materials and metal materials, combined, have the potential worth developing in the application of SERS substrates and certain catalytic reactions.

## 2. Experimental

### 2.1. Chemicals

All the chemicals were purchased from Sinopharm Chemical Reagent Co. Ltd. (Shanghai, China) and applied directly without any treatment. The aqueous solutions involved are freshly prepared from Milli-Q purified water (>18 MΩ cm^−1^).

### 2.2. Instrumentation

Scanning electron microscopy (SEM) images were collected on a Hitachi SU8010 field emission scanning electron microscope (Tokyo, Japan) at 10 kV. Transmission electron microscope (TEM) and high resolution transmission electron microscopy (HRTEM) images were recorded by using a JEM-2100 ultrahigh-resolution transmission electron microscope (Tokyo, Japan) at 200 kV. X-ray photoelectron spectroscopy (XPS) studies were carried out on the thermo scientific ESCALAB 250 instrument (Waltham, MA, USA) using an Al Kα monochromated (150 w, 30.0 eV Pass Energy, 500 µm Spot Size). X-ray diffraction (XRD) patterns have been recorded on a Bruker D8 ADVANCE (Brooke, Germany) diffractometer by employing Cu Kα radiation. SERS images were captured using a Renishaw (Renishaw, UK) inVia Reflex confocal Raman system with an excitation wavelength of 532 nm and a power of 5 mW. The spectral range is 200–1000 nm and the spectral resolution is 1 cm^−1^. Spectra were collected by focusing the laser line onto the sample using a 50 × (numerical aperture of 0.75) objective, providing a spatial resolution of approximately 1 mm. The data acquisition time was 1 s for a single accumulation.

### 2.3. Preparation of Ag–ZnO Nanocomposite

A uniform zinc oxide nanorod array was prepared by the previous method [27,28]. First, zinc acetate was dissolved in a mixed solution of ethanolamine and 2-methoxyethanol, where the concentrations of zinc acetate and ethanolamine are both 0.75 M. The mixed solution was heated and stirred for 30 min at 60 °C to obtain ZnO seed solution. Thereafter, the 1 × 2 silicon wafer was immersed in the above-mentioned seed solution, and then taken out after 30 min to dry and then thermally annealed for 10 min at 300 °C. The annealed silicon wafer was immersed in a mixed solution of 0.1 M zinc nitrate and hexamethylenetetramine, and hydrothermally heated for 2 h at 95 °C. Finally, the silver nanoparticles were grown on the ZnO nanorod array by chemical reduction and impregnation. The specific process entailed immersing the ZnO array substrate in 0.1 M NaBH_4_ for 5 s, and then immersing it in 0.1 M AgNO_3_ solution for 5 s. We repeated the process four times, then rinsed the substrate with deionized water and blew it dry with nitrogen.

## 3. Results and Discussion

### 3.1. Characterization of Ag–ZnO Nanocomposite

**Figure 1:**
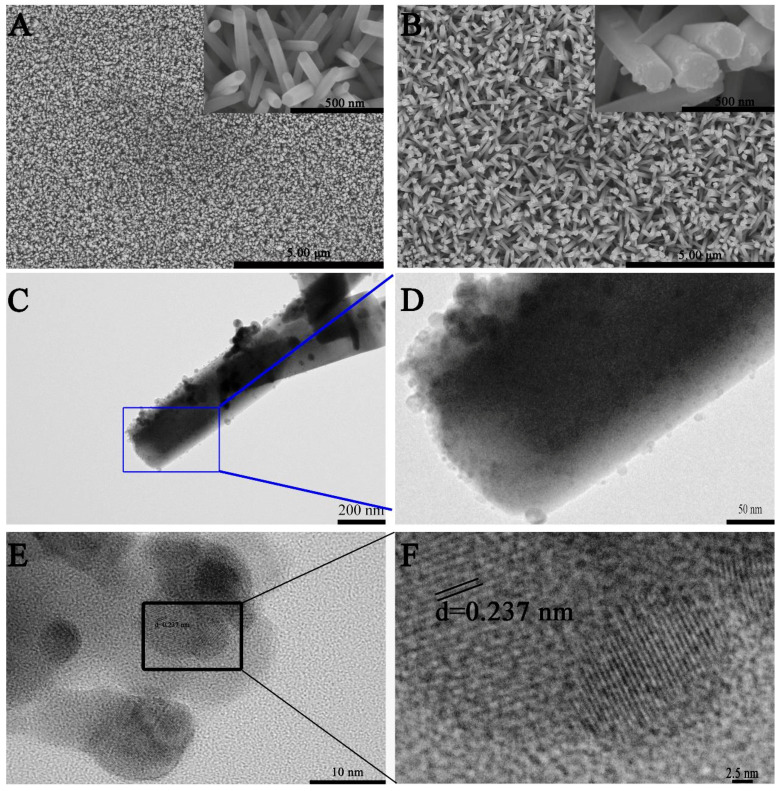
(**A**) SEM image of the pure ZnO nanorod and (**B**) Ag–ZnO nanorod array; (**C**) TEM image of the Ag–ZnO nanorod array and (**D**) partial enlarged view of (**C**,**E**) HRTEM image of the Ag–ZnO nanorod array and (**F**) partial enlarged view of (**E**).

As shown in Figure 1A, the SEM image shows that subsequent to thermal annealing and hydrothermal growth, ZnO nanorods with a diameter of about 150 nm are uniformly grown on the silicon wafer. Subsequent to a simple chemical reduction immersion method, the silver nanoparticles with an average diameter of about 25 nm have been successfully grown on the ZnO nanorods, as shown in the Figure 1B. Further, Figure 1C,D are the TEM image of Ag–ZnO composite material, and it can be clearly seen that the silver nanoparticles are arranged evenly and densely on the ZnO nanorods (Figure 1D is a partial enlarged view of Figure 1C). The HRTEM images of the composite material have been shown in Figure 1E,F, and the lattice spacing of 0.237 nm corresponds to the (111) plane of the silver nanoparticles. Therefore, it can be proved that the silver nanoparticles have been successfully grown on the ZnO nanorods. (Figure 1F is a partial enlarged view of Figure 1E).

**Figure 2:**
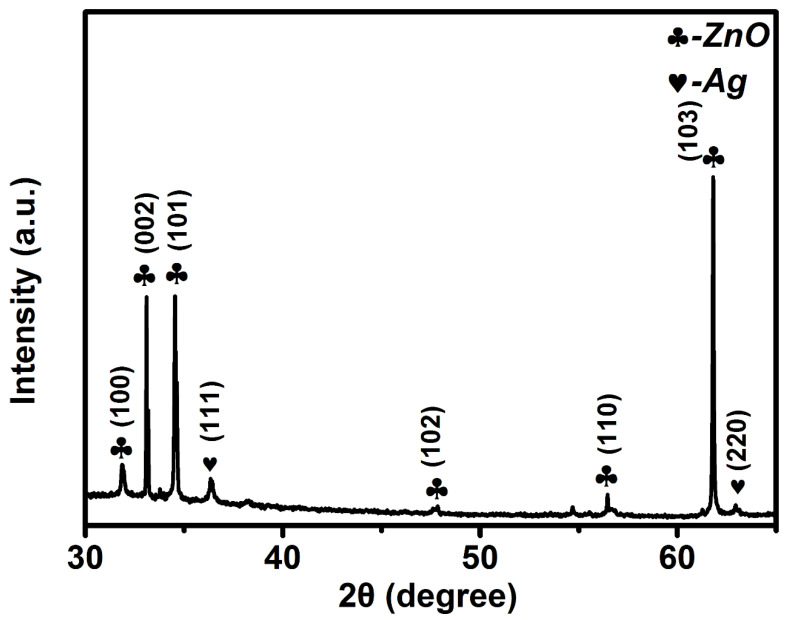
XRD spectra of the Ag–ZnO nanorod array.

Figure 2 shows the XRD pattern of the nanocomposite material. The diffraction patterns have been indexed with Bragg planes (100), (002), (101), (102), (110), (103) and (111), (220) following the comparison with standard pattern, suggesting that both wurtzite ZnO and metal Ag exist and confirming the successful preparation of Ag–ZnO nanocomposites.

**Figure 3:**
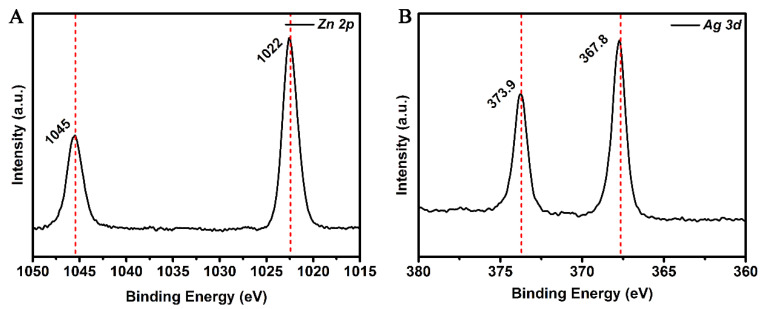
(**A**)XPS spectra of Zn 2p; (**B**) Ag 3d.

Figure 3A shows the XPS spectrum of Zn2p in the Ag–ZnO nanocomposite. It can be seen from the spectrum that it presents two independent symmetrical peaks. The two characteristic peaks with binding energies at 1023 and 1046 eV have been attributable to Zn 2p_3/2_ and Zn 2p_1/2_, respectively, indicating that Zn mainly exists in the form of Zn^2+^ [29]. Figure 3B shows the XPS spectrum of Ag 3d, where the two characteristic peaks at 367.8 and 373.9 eV have been attributed to Ag 3d_3/2_ and Ag 3d_5/2_, respectively [30,31]. It can be seen that the binding energy of Ag 3d in the composite material moves to a high field, indicating that the electron transfer has occurred between Ag–ZnO (BE value of Ag^0^ and Ag^+^ is about 368.2 and 367.2 eV, respectively). Since the Fermi energy level of Ag is higher than ZnO, a Mott Schottky barrier will be formed at the Ag–ZnO interface, and the hot electrons will be transferred from Ag to the conduction band of ZnO until the Fermi energy levels of both sides are in a straight line. Consequently, the number of hot electrons on the Ag surface decreases.

### 3.2. Ag–ZnO as the SERS Substrate

**Figure 4:**
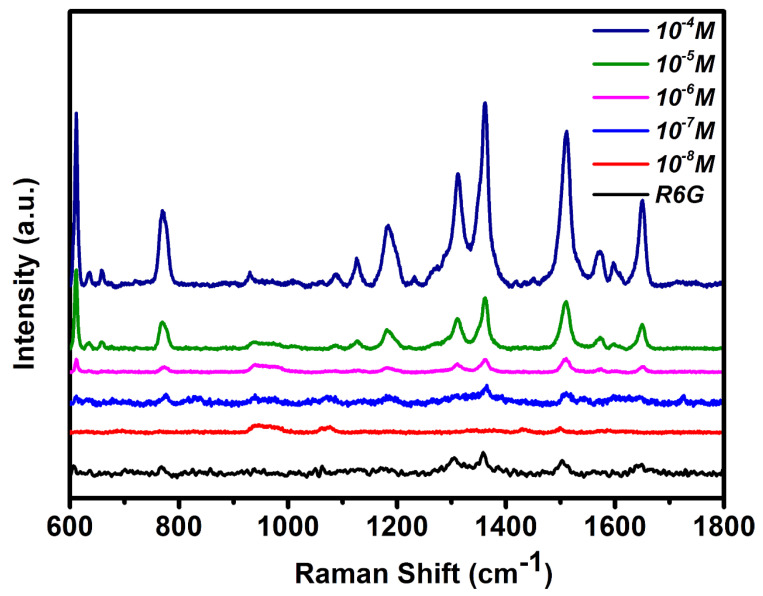
SERS images of 10^−4^–10^−8^ M R6G adsorbed on the Ag–ZnO nanorod array substrate.

To study the feasibility of Ag–ZnO composite nanomaterials as SERS substrates, R6G probe molecules have been used to test their SERS enhancement performance. Figure 4 shows the Raman spectrum of R6G molecules with concentrations ranging from 10^−4^ to 10^−8^ M adsorbed on the Ag–ZnO substrate, the peak at 611 cm^−1^ assigned to C–C–C bond stretching vibration, the peaks at 1309, 1365, 1509, the peak of 1647 cm^−1^ assigned to C-C stretching modes, the peak at 770 cm^−1^ assigned to the out-of-plane vibration of deformed C-H bonds, and the peak at 1573 cm^−1^ assigned to C-O-C bond stretching vibration. It can be seen that the characteristic Raman peak of R6G molecules can still be observed when the concentration is as low as 10^−8^ M.

### 3.3. Coupling Reaction of PATP on Ag–ZnO Substrate

**Scheme 1:**
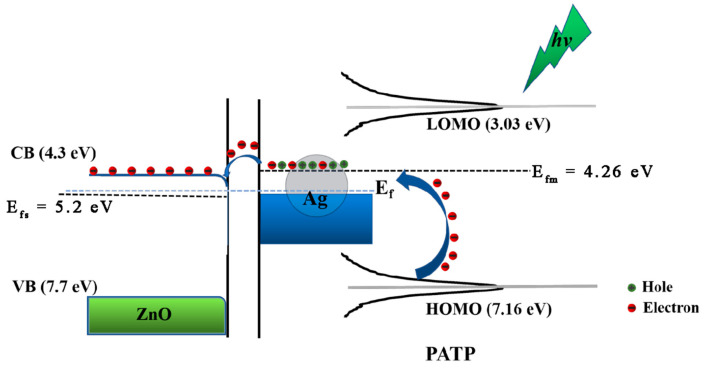
Schematic diagram of the coupling reaction mechanism of PATP adsorbed on Ag–ZnO substrate.

**Figure 5:**
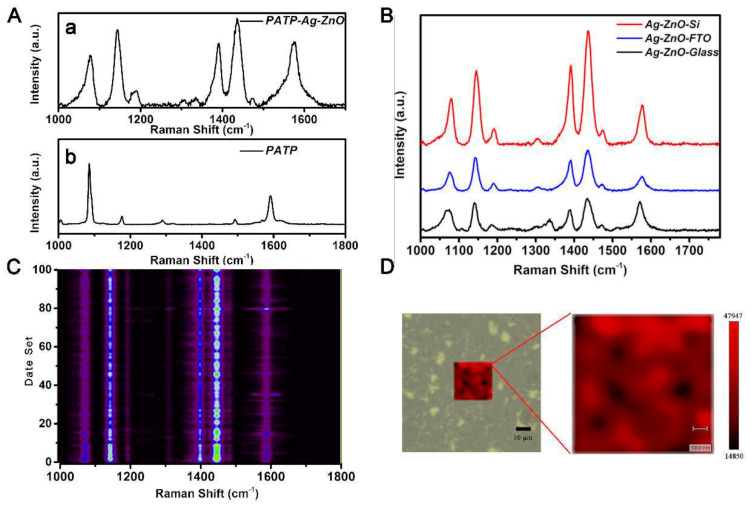
(**A**) a—SERS spectra of 10^−5^ M PATP on the Ag–ZnO–Si substrate, b—SERS spectra of PATP powder; (**B**) SERS spectra of 10^−5^ M PATP on Ag–ZnO substrate supported by different substrates; (**C**) Raman mapping images of 10^−5^ M PATP adsorbed on the Ag–ZnO nanorod array substrate; (**D**) Raman intensity distribution map at 1434 cm^−1^ in the selected range.

Figure 5A shows the Raman mapping of 10^−5^ M PATP adsorbed on the Ag–ZnO substrate, and the three characteristic peaks of 1140, 1390, and 1434 cm^−1^ belonging to DMAB can be clearly observed, indicating the occurrence of the coupling reaction of PATP molecules adsorbed on Ag–ZnO substrate. Table 1 shows the measured frequencies of the Raman bands and their assignments for PATP and DMAB. The coupling reaction of PATP is an oxidation reaction induced by thermal holes [32]. The Fermi level of Ag is higher than that of ZnO, and a Mott Schottky barrier is formed at the Ag–ZnO interface. The hot electrons have been transferred from Ag to the conduction band of ZnO until the Fermi levels of both sides are at the same level [26,33]. The number of hot electrons on the Ag surface decreases, and the hot holes increase. This conclusion has been confirmed by the previous XPS conclusion. Concomitantly, silver nanoparticles absorb light and generate electron-hole pairs through the surface plasmon activity. The thermal pores in the metal can capture electrons from the adsorbed PATP molecule HOMO. The combined effect of the charge transfer and surface plasmon promotes the PATP coupling reaction (Scheme 1).

To eliminate the influence of the supporting substrate on the experimental results, we have used Silicon wafer, ordinary glass, and conductive glass as the support 10^−5^ M PATP as the probe molecule, and obtained Raman spectra under the same experimental conditions (Figure 5B). It can be seen that the PATP coupling reaction occurs on different supports, indicating that the support has no effect on the reaction. A total of 100 samples in a 10 × 10 μm square area on the substrate have been selected for the mapping analysis. As shown in Figure 5C,D, it can be seen that the reaction has a good response effect in the selected area. It shows that the substrate has an excellent uniformity and good Raman enhancement effect.

## 4. Conclusions

To summarize, the Ag–ZnO nanorod array has been successfully synthesized. When it is used in a SERS substrate, it exhibits an excellent enhancement effect, and the uniformity of the substrate is confirmed with the use of the mapping technology. Since the Fermi level of Ag is higher than that of ZnO and the Mott Schottky barrier formed at the Ag–ZnO interface, the electron transfer will occur between ZnO–Ag until the Fermi level reaches a certain level. This electron transfer, together with the surface plasma, promotes the coupling reaction of PATP molecules. The nanocomposite material combined with the semiconductor and metal nanoparticles has a great potential advantage as a uniform and stable SERS substrate with a good enhancement effect. Concomitantly, owing to the special properties of the charge transfer between the semiconductor materials and metal nano-particles, it also has certain potential applications in the field of catalysis.

## Figures and Tables

**Table 1:** The measured frequencies of the Raman bands and their assignments for PATP and DMAB.

PATP		DMAB	
Assignment	Measured Frequency (cm^−1^)	Assignment	Measured Frequency (cm^−1^)
N(CS) + N(CC)	1087	ν(CN) + δ(CH)	1146
Δ(CH)	1179	ν(NN) + ν(CC) + δ(CH)	1390
N(CC) + Δ(CH)	1289	ν(NN) + δ(CH) + ν(CC)	1435
N(CC) + Δ(CH)	1493		
N(CC) + Δ(NH2) + Δ(CH)	1592		

## Data Availability

Data is contained within the article.
